# Association between solid fuel use and cognitive decline among middle-aged and elderly Chinese adults: a longitudinal study

**DOI:** 10.1038/s41598-021-83171-7

**Published:** 2021-02-11

**Authors:** Haoqiang Ji, Liang Du, Meng Sun, Yuxin Duan, Jia Xu, Ruiheng Wu, Xu Chen, Yuanping Pan, Yunting Chen, Ling Zhou

**Affiliations:** School of Public Health, Dalian Medical University, 9 Western Section, Lvshun South Street, Lvshunkou District, Dalian, People’s Republic of China

**Keywords:** Cognitive ageing, Public health, Environmental impact

## Abstract

This study was conducted to investigate (1) the association between solid fuel use for cooking and cognitive function; (2) the moderating effects of gender and residential area on cognitive scores among solid fuel users; and (3) the effects of solid fuel use on cognitive decline among different gender and age subgroups in 2011–2018. A total of 5140 Chinese middle-aged and elderly participants were successfully followed for 7 years (2011–2018). Solid fuel use was self-reported as using solid fuel for cooking at home, and cognitive function was assessed by 4 parts: episodic memory, time orientation, numerical ability and picture drawing. After adjusting for covariates, solid fuel users had lower cognitive scores, and the moderation effects of gender and residence on cognitive function were significant among the solid fuel users. In addition, compared with the group of clean fuel users, solid fuel users had a faster decline rate of cognitive function among the subgroups of female and elderly people.

## Introduction

In developing countries, indoor air pollution (IAP) is one of the leading causes of several diseases and premature death^1^. As the World Health Organization (WHO) reports, there are 3.8 million premature deaths annually in low- and middle-income countries that may be attributed to IAP from cooking fires^2^. Even so, approximately 2.7 billion individuals who lacked access to clean cooking facilities used solid fuel (coal, firewood, animals dung) to meet the most basic energy needs in developing countries, mainly referring to cooking in 2018^3^. In these households, solid fuels are usually burned in inefficient or poorly ventilated combustion devices (traditional stoves or open fires)^4^. The incomplete combustion of solid fuels can exhaust many potentially toxic pollutants, including particulate matter (PM), carbon monoxide (CO), nitrogen dioxide and other air pollutants^5^. While using solid fuels in cooking, a high-level dosage of PM is exhausted (1181.4–5891.7 µg/m^3^), which is higher than that of other fuels^6^. Once PM is inhaled by the body, it can travel through our circulatory system and eventually damage organs, such as the lungs, heart, and even the brain, which may cause brain inflammation and neuronal dysfunction^7,8^.

In numerous studies, the mixture of pollutants from burning solid fuel was certified as the cause of several diseases, such as cardiovascular disease^9^, acute respiratory infections^10^, low birth weight^11^, chronic obstructive pulmonary disease, lung cancer, tuberculosis^12^, asthma^13^, and diseases of eye, but few studies have investigated its role in cognitive decline. Currently, most studies have primarily paid attention to the association between outdoor air pollution and cognitive decline, such as ambient air pollution^14,15^ and traffic-related air pollution^16,17^. Only a few studies of IAP have suggested that household incense burning causes cognitive decline^18,19^ and one cross-sectional study showed that there is an association between solid fuel use and cognitive function^20^.

China is one of the largest developing countries with a population of 1.4 billion. Most populations are threatened by IAP, with widespread use of solid fuel^3^. In China, fuels for household use contributed only 7.5% of the total Chinese energy consumption but contributed 71% and 27% of the indoor and outdoor PM2.5 concentrations, respectively, and 67% of PM2.5-related premature deaths in 2014^21^. Meanwhile, China is also one of the largest aging countries, and the aging problem will reach its peak after a few decades^22^. The cognitive decline of middle-aged and elderly people is one of the most important public health problems. There is a growing amount of evidence showing that cognitive decline can be affected by many factors, and cognitive decline affects both elderly individuals and middle-aged individuals^23,24^ because they all face the constant effects of aging, other diseases and environmental threats. Under this background, IAP from burning solid fuel may have more long-lasting and serious effects on cognitive decline among these people^25,26^. Taking into account the rapid growth of the middle-aged and elderly populations and the prevalence of cognitive deficits, further evidence about the association between solid fuel use and cognitive function is invaluable in China.

However, previous studies linking solid fuel use to cognitive decline lacked enough evidence, especially longitudinal studies^19,20^. In this study, we used a nationally representative sample of Chinese middle-aged and elderly participants to explore the hypothesis that solid fuel use for cooking is associated with worse cognitive function and a faster cognitive decline rate and to assess the moderation effect of gender and residential area on cognitive function among solid fuel users.

## Methods and measurements

### Study sample

**Figure 1:**
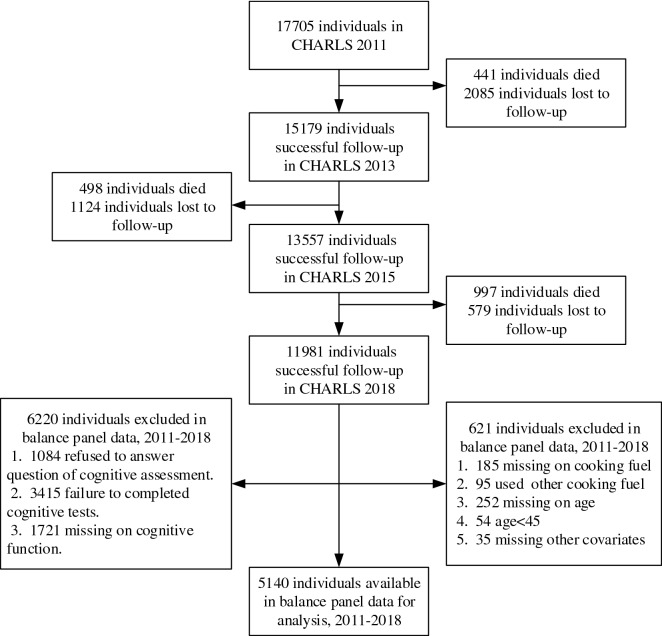
Flowchart of participant eligibility in the current study.

The China Health and Retirement Longitudinal Study (CHARLS) is a nationally representative longitudinal survey of persons in China 45 years of age or older that was conducted by Peking University. The baseline survey was selected from 23 Chinese provinces using multistage sampling in 2011, and 3 follow-up surveys were conducted in 2013, 2015 and 2018. Details of the design and methodology regarding this program have been described elsewhere^27^. The CHARLS datasets from the 4 survey waves were used in the study. There were 17,705 respondents at baseline, and 11,981 of them continued to take the survey from 2011 to 2018 after excluding those lost to follow-up and the deceased population. Furthermore, we removed the records that failed to meet the research requirements, which led to 5140 eligible respondents in the study. Figure 1 shows the process of exclusion of research participants in the study.

### Cognitive assessment

Cognitive function was assessed by four composite measurements, including episodic memory, time orientation, numerical ability and picture drawing^27^. First, to measure episodic memory, interviewers read 10 Chinese words and asked participants to repeat the words that they remembered (immediate recall). In addition, they were asked to recall the 10 words 5 min later (delayed recall) and were given 1 point for each word they recalled correctly. The score for episodic memory was equal to the average of the sum of immediate recall and delayed recall, ranging from 0 to 10. Second, the measurement of time orientation required respondents to recall today’s date (year, month, day), the day of the week, and the current season. The time orientation score was equal to the number of correct answers to the 5 questions, ranging from 0 to 5. Third, the numerical ability required respondents to perform 7 serial subtractions starting from 100 (up to 5 times), and the total score was equal to the number of correct calculations, ranging from 0 to 5. However, this total score was reduced by half if participants used paper, pen or other aid to complete this assessment. Fourth, to measure the ability to draw a picture, interviewers showed a picture of 2 pentagons overlapping with participants and asked them to draw this picture on a white paper. The respondents who successfully drew this picture received 1 point, but those who failed to draw it received no score. Finally, the total cognitive function score was defined as the sum of all 4 parts, ranging from 0 to 21^28^.

### Solid fuel exposure

Participants’ exposure to cooking fuels was assessed by an item of the questionnaire. The interviewer asked the respondents the question “what is the main source of cooking fuel?”, and the response options included coal, crop residue, wood, natural gas, marsh gas, liquefied petroleum gas, electric, never cook and others. Because we were not sure what the other fuel was, we excluded the users of other fuels and those that answered that they never cook. Considering a previous study on the health effects of fuel burning^21^, we defined cooking fuel as solid fuel (coal, crop residue and wood) and clean fuel (natural gas, marsh gas, liquefied petroleum gas and utilizing electricity).

### Covariates

We controlled for 4 sets of potential covariates associated with cognition and solid fuel use in the study, including sociodemographic characteristics, health behaviors, health status and time (years since baseline). The sociodemographic characteristics included gender (male/female), age (continuous), residential area (rural/urban), marital status (married/unmarried), and education (no finished primary school/primary/middle school/high school and above). The health behaviors included smoking status (never smoker/current smoker/former smoker), drinking status (never drinker/a little/frequency), and sleep time (< 6 h/6–8 h/> 8 h). Health status comprised depressive symptoms (no/yes, assessed by CESD-10)^28^, number of chronic diseases (0/1/≧ 2, diagnosed by a doctor), and self-rated health (good/fair/poor, assessed by themselves).

### Statistical analysis

First, we used multiple imputation (MI) to estimate the missing values for depressive symptoms. We used the linear regression model based on gender, age, residential area, education level, marital status, chronic diseases, self-rated health and cognitive score as predictors to estimate 20 replications to account for missing depressive scores per year. Second, baseline characteristics of CHARLS are described as the mean ± standard deviation (SD) for the continuous variables or percentages (%) for categorical variables according to cooking fuel use. T tests (for continuous variables) and Pearson’s chi-square (for categorical variables) were applied to compare the differences in basic characteristics between the solid fuel users and clean fuel users. Third, considering the variations in participant characteristics over time and the lack of independence of repeated measurement data, we used a linear mixed model to verify the hypotheses of the study. After adjusting for gender, age, residential area, marital status, education, smoking status, drinking status, sleep time, depressive symptoms, chronic diseases, self-rated health, cooking fuel use × time and time (years since baseline), we used a linear mixed model to assess the effect of solid fuel use on cognitive function. Fourth, to assess the moderation effect of gender and residential area, we added interaction terms to the linear mixed models. Finally, to examine the difference in the decline rate of the cognitive score among the different groups, the study, based on gender and age stratification, added an interaction term for cooking fuel use and time to the linear mixed models with adjustment for all covariates. All data processing and analyses were performed in STATA version 14.0 (StataCorp LLC, College Station, Texas, USA). All tests were two-sided, and *P* < 0.05 was considered statistically significant. The analysis results for the linear mixed models are reported using standardized coefficients (β) and their 95% confidence intervals (CIs).

## Results

### Baseline characteristics

A total of 5140 participants were included in the final analyses; the mean age was 58.18, and 52.2% of the participants were men. A total of 2420 (47.1%) participants used solid fuel for cooking, and 1618 (34.5%) respondents still used it in 2018. Most of the participants (77.4%) lived in rural areas, and 2565 (64.5%) reported using solid fuel for cooking. Approximately one-third of the participants had not finished primary school, and 66.6% of these participants used solid fuel. Furthermore, compared with clean fuel users, solid fuel users were more likely to be women, older, live in rural areas, not have finished primary school, current smoker, have a sleep time less than 6 h, depressive symptoms, 2 or more chronic diseases, and a poor health status and lower cognitive scores (*P* < 0.01 for all). Table 1 shows more of the baseline characteristics according to different cooking fuel uses.

**Table 1:** Baseline characteristics of study participants.

Characteristics	Total (N = 5140)	Clean fuel (N = 2720)	Solid fuel (N = 2420)	*P* value
Cognition (mean ± SD)	12.40 ± 3.74	12.72 ± 3.67	11.19 ± 4.11	< 0.001
Age (mean ± SD)	58.18 ± 8.24	61.27 ± 8.74	61.64 ± 8.50	0.002
**Gender (%)**				0.215
Male	2685 (52.2)	1242 (46.3)	1443 (53.7)	
Female	2455 (47.8)	1178 (48.0)	1277 (52.0)	
**Residential area (%)**				< 0.001
Rural	3978 (77.4)	1413 (35.5)	2565 (64.5)	
Urban	1162 (22.6)	1007 (86.7)	155 (13.3)	
**Marital status (%)**				0.833
Married	4538 (88.3)	2139 (47.1)	2399 (52.9)	
Unmarried	602 (11.7)	281 (46.7)	321 (53.3)	
**Education level (%)**				< 0.001
Unfinished primary school	1552 (30.2)	519 (33.4)	1033 (66.6)	
Primary school	1321 (25.7)	563 (42.6)	758 (57.4)	
Middle school	1447 (28.2)	768 (53.1)	679 (46.9)	
High school or above	820 (15.9)	570 (69.5)	250 (30.5)	
**Smoking status (%)**				< 0.001
Never smoker	3031 (59.0)	1508 (49.8)	1523 (50.2)	
Current smoker	1650 (32.1)	704 (42.7)	946 (57.3)	
Former smoker	459 (8.9)	208 (45.3)	251 (54.7)	
**Drinking status (%)**				0.495
Never drinker	3326 (64.7)	1562 (47.0)	1764 (53.0)	
A little	1363 (26.5)	634 (46.5)	729 (53.5)	
Frequency	451 (8.8)	224 (49.7)	227 (50.3)	
**Sleep time (%)**				< 0.001
< 6 h	1357 (26.4)	564 (41.6)	793 (58.4)	
6–8 h	3389 (65.9)	1685 (49.7)	1704 (50.3)	
> 8 h	394 (7.7)	171 (43.4)	223 (56.6)	
**Depressive symptom (%)**				< 0.001
No	3480 (67.7)	1834 (52.7)	1646 (47.3)	
Yes	1660 (32.3)	586 (35.3)	1074 (64.7)	
**Chronic diseases (%)**				0.005
0	1750 (34.1)	860 (49.1)	890 (50.9)	
1	1523 (29.6)	736 (48.3)	787 (51.7)	
2 or above	1867 (36.3)	824 (44.1)	1043 (55.9)	
**Self-rated health (%)**				< 0.001
Good	1436 (27.9)	794 (55.3)	642 (44.7)	
Fair	2658 (51.7)	1265 (47.6)	1393 (52.4)	
Poor	1046 (20.4)	361 (34.5)	685 (65.5)	

### Association between cooking fuel use and cognitive function

In the multivariate analysis of the linear mixed model, compared with clean fuel users, solid fuel users had lower cognitive scores (β =  − 0.17, *P* = 0.001). Moreover, gender, age, residential area, marital status, educational level, drinking status, sleep time, depressive symptoms, chronic diseases, self-rated health, time, the interaction of time and cooking fuel use all had a significant relationship with cognitive function (*P* < 0.05 for all). Table 2 describes more details about the factors influencing cognitive function.

**Table 2:** Association of cognitive function with cooking fuel use.

Characteristics	Groups	β	95%CI	*P* value
Cooking fuel use	Clean fuel	Reference	Reference	Reference
	Solid fuel	− 0.17	− 0.04, − 0.01	0.001
Gender	Male	Reference	Reference	Reference
	Female	− 0.75	− 0.91, − 0.58	< 0.001
Age		− 0.06	− 0.07, − 0.05	< 0.001
Residential area	Rural	Reference	Reference	Reference
	Urban	0.86	0.72, 0.99	< 0.001
Marital status	Married	Reference	Reference	Reference
	Unmarried	− 0.42	− 0.56, − 0.28	< 0.001
Education level	Unfinished primary school	Reference	Reference	Reference
	Primary school	1.83	1.68, 1.97	< 0.001
	Middle school	2.69	2.52, 2.85	< 0.001
	High school or above	3.44	3.23, 3.65	< 0.001
Smoking status	Never smoker	Reference	Reference	Reference
	Current smoker	− 0.11	− 0.23, 0.01	0.08
	Former smoker	0.07	− 0.07, 0.22	0.33
Drinking status	Never drinker	Reference	Reference	Reference
	A little	0.22	0.07, 0.36	0.003
	Frequency	0.03	− 0.09, 0.15	0.596
Sleep time	< 6 h	Reference	Reference	Reference
	6–8 h	0.02	− 0.07, 0.11	0.605
	> 8 h	− 0.39	− 0.55, − 0.23	< 0.001
Depressive symptom	No	Reference	Reference	Reference
	Yes	− 0.76	− 0.85, − 0.67	< 0.001
Chronic diseases	No	Reference	Reference	Reference
	1	0.05	− 0.06, 0.15	0.15
	2 or above	0.13	0.02, 0.24	0.02
Self-rated health	Good	Reference	Reference	Reference
	Fair	− 0.11	− 0.21, − 0.01	0.025
	Poor	− 0.49	− 0.63, − 0.36	< 0.001
Time		− 0.03	− 0.05, − 0.01	0.006
Solid fuel use × time		− 0.03	− 0.06, − 0.01	0.02

To analyze the moderation effect of gender, an interaction term was added to the linear mixed model. Compared with the reference, female users of solid fuel had lower cognitive scores (β =  − 0.96, *P* < 0.001), but there was no significant interaction between male gender and solid fuel users. In addition, in the model assessing the moderation effect of residential area, solid fuel users in rural areas had lower cognitive scores (β =  − 1.01, *P* < 0.001), but no interaction was not found for solid fuel users in urban areas (Table 3).

**Table 3:** The interaction between cooking fuel use and socio-demographic characteristics.

Interaction term	β	95% CI	*P* value
**Cooking fuel use × gender**			
Clean fuel × male	Reference	Reference	Reference
Clean fuel × female	− 0.66	− 0.84, − 0.48	< 0.001
Solid fuel × male	− 0.06	− 0.22, 0.09	0.405
Solid fuel × female	− 0.96	− 1.17, − 0.75	< 0.001
Time	− 0.03	− 0.05, − 0.01	0.002
Solid fuel × time	− 0.03	− 0.06, − 0.01	0.017
**Cooking fuel use × residential area**			
Clean fuel × urban	Reference	Reference	Reference
Clean fuel × rural	− 0.84	− 0.99, − 0.69	< 0.001
Solid fuel × urban	− 0.26	− 0.54, 0.02	0.069
Solid fuel × rural	− 1.01	− 1.18, − 0.83	< 0.001
Time	− 0.03	− 0.05, − 0.01	0.003
Solid fuel × time	− 0.03	− 0.06, − 001	0.017

In the linear mixed model of assessing the moderation effect of gender, we adjusted for cooking fuel use, time, cooking fuel use × time, age, residential area, marital status, education, smoking status, drinking status, sleep time, depressive symptom, chronic diseases and self-rated health. Besides, to analysis the moderation effect of residential area, we adjusted for cooking fuel use, time, cooking fuel use × time, age, gender, marital status, education, smoking status, drinking status, sleep time, depressive symptom, chronic diseases and self-rated health.

### The decline of cognitive function from 2011 to 2018

**Figure 2:**
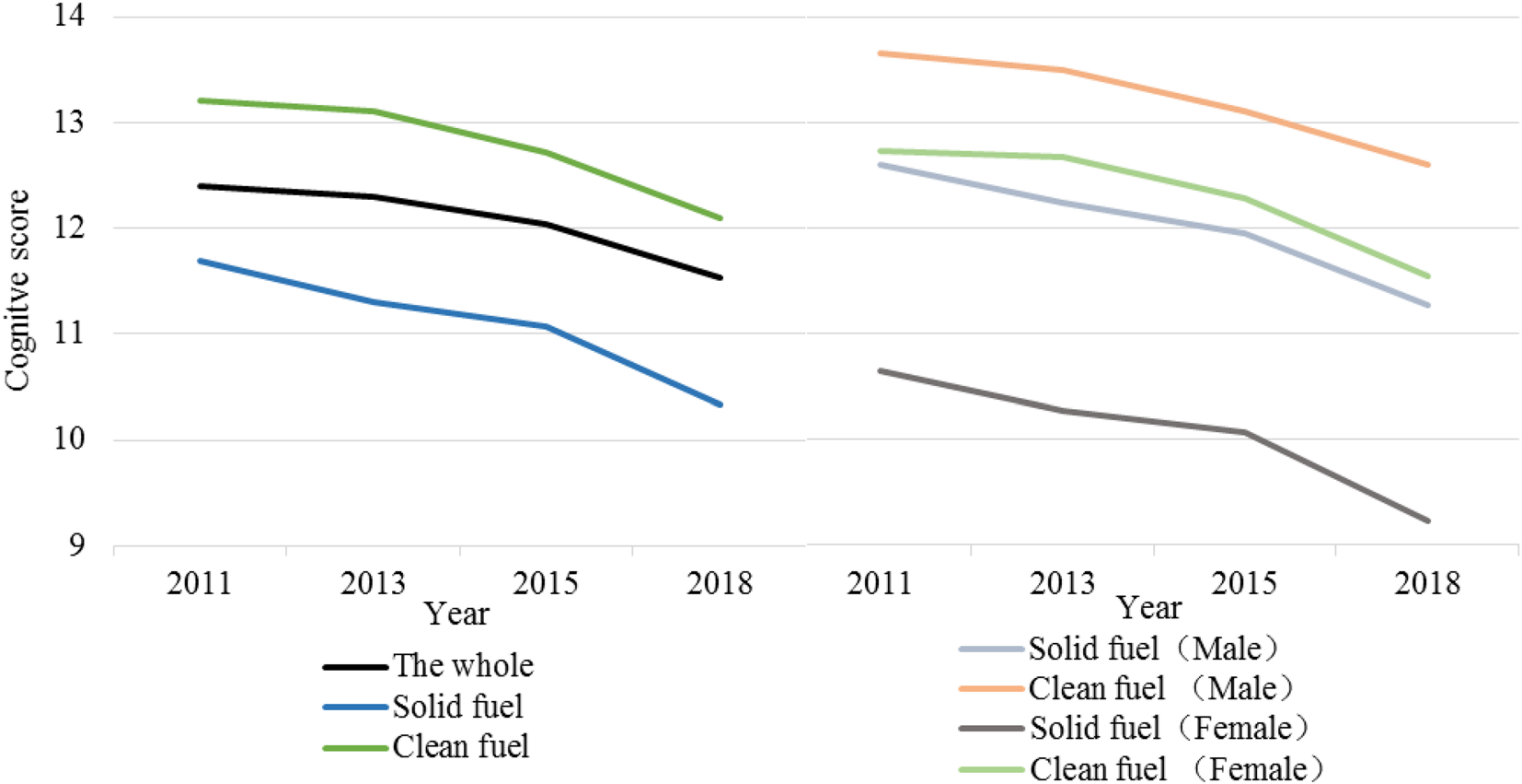
Cognitive change in the participants during the follow-up period.

During the 7-year follow-up period, the cognitive scores of the different groups continued to drop. Compared with clean fuel users, participants who used solid fuel for cooking had a faster decline rate in cognitive scores (from 11.7 to 10.3, annual decline rate: 1.8%). In addition, in the follow-up period, the cognitive scores of male users of clean fuel fell slower than those of the other groups (from 13.7 to 12.6, annual decline rate: 1.2%), and female users of solid fuel had the fastest decline rate (from 10.7 to 9.2, annual decline rate: 2.1%) (Fig. 2).

### Association between solid fuel use and the rates of cognitive decline

In the linear mixed models, there were different decline rates of cognitive function in the different subgroups. In the total sample, female sample and rural samples, solid fuel users had faster decline rates of cognitive scores (*P* < 0.05 for all), but the same effect was not found in the male (*P* = 0.147) or middle-aged samples (*P* = 0.719). Compared with the reference groups, the decline rate of the cognitive scores increased by 0.04 units per year (β =  − 0.04; 95% CI − 0.09, 0.01) among female users and 0.06 units among the elderly sample (β =  − 0.06; 95% CI − 0.11, 0.01) (Table 4).

**Table 4:** Association between solid fuel use and the rate of cognitive decline.

Groups	Solid fuel × time (β)	95% CI	*P* value
Total sample	− 0.03	− 0.06, − 0.01	0.017
Male sample	− 0.03	− 0.07, 0.01	0.147
Female sample	− 0.04	− 0.09, − 0.01	0.041
Middle-aged sample (45 ≦ age < 65)	0.01	− 0.03, 0.04	0.719
Elderly sample (age ≧ 65)	− 0.06	− 0.11, − 0.01	0.013

In liner mixed models of different gender subgroups, we adjusted for cooking fuel use, time, cooking fuel use × time, age, residential area, marital status, education, smoking status, drinking status, sleep time, depressive symptom, chronic diseases and self-rated health. In age subgroups, we adjusted for cooking fuel use, time, cooking fuel use × time, gender, residential area, marital status, education, smoking status, drinking status, sleep time, depressive symptom, chronic diseases and self-rated health.

## Discussion

As one of the largest prospective cohort studies in Chinese adults, this study found that solid fuel users had poorer cognitive function, especially female users and rural users. In addition, compared with clean fuel users, solid fuel users had a faster decline rate of cognitive function, especially among the female and older samples. Therefore, gender inequality and rural–urban differences deserve more attention, especially in the elderly.

The study is in line with several previous studies. A cross-sectional study that included 13,023 older (age 50+) Mexican adults examined whether exposure to indoor air pollution from cooking fuel (coal or wood) was associated with poorer cognitive function^20^. In addition, Qiu et al. found a significant adverse impact of IAP use for cooking on cognitive abilities in Chinese middle-aged and elderly people, specifically short-term memory and mathematical reasoning^19^. However, these previous studies used a cross-sectional study design, and therefore our study supplemented the results from a longitudinal study and found a significant association between solid fuel use and cognitive decline (especially in the female and elderly samples). Our study may help develop intervention strategies for the rapidly increasing aging population and environmental pollution in China.

Although we found that solid fuel use was related to slight cognitive decline, the continuous adverse health impacts cannot be ignored for middle-aged and elderly people^29^. The evidence about the mechanism of solid fuel use exposure-related cognitive decline is not extensive, but it may be linked to the fine PM, CO or other pollutants released by solid fuel combustion^30^. The burning of solid fuels produces high concentrations of PM and other pollutants, which may increase brain inflammation and the accumulation of β-amyloid, a marker of neuronal dysfunction^8^. Therefore, individuals who suffer from PM pollution may be more likely to develop cognitive deficits, structural brain aging and even neurodegenerative diseases^31,32^. The elderly are especially more susceptible to various environmental risk factors, such as PM-burning solid fuel^23^. If cognitive function continues to decline, it will influence the normal life of people and cause Alzheimer’s disease^25^. Even so, in parts of the Chinese countryside, where rapid economic growth and infrastructure expansion have contributed to universal access to electricity, solid fuel use has persisted^3^, which may cause additional health hazards to the rural population. In summary, the use of solid fuel for cooking not only causes disease suffering and cognitive decline but also exacerbates aging and environmental pollution problems in China.

Solid fuel use not only brings about severe health risks to users but also exacerbates gender inequality in a negative way^33,34^. In low- and middle-income regions such as rural China, women have lower cognitive function than men because families may only emphasize the development of male ability traditionally but ignore women’s demands^35^. Just so, it is difficult for women to improve their cognitive ability in the course of personal development^36^. In addition to the lower cognitive function, female users of solid fuel for cooking had a faster decline rate of cognitive function than male users. Women usually take care of their family and cooking, whereas men traditionally work away from home during the day^37^. The Chinese tradition leads to women being particularly exposed to IAP from solid fuel burning and having a higher risk of developing IAP-associated adverse health conditions^38–40^. Therefore, solid fuel use and Chinese tradition bring about health impacts and gender inequality that are too significant to be ignored among the middle-aged and elderly.

Overall, necessary steps should be taken in China to alleviate the impact of solid fuel use on health. Promoting cleaner fuels, using stoves with chimneys and improving ventilation efficiency may be the best way to reduce the exposure of IAP within households, but it is a challenge that clean fuels are widely used for cooking in remote rural areas^41^. Poorer families are likely to stick with solid fuels for cooking in rural areas because alternative clean fuels cannot be affordable for them, and some biomass fuels are everywhere in the countryside, such as straw, animal dung and others. Governments must consider the personalized needs of these people and work with community leaders to implement programs about reducing exposure to IAP caused by solid fuel burning^20^. In addition, other developmental objectives, such as promoting entrepreneurship, providing jobs and women’s empowerment, also reduce the exposure of burning solid fuel, especially for women^42^. After all, women benefit more from fruits of economic development—it may less female disadvantage in cognitive function^35^.

This study has several limitations. First, our study results can only be generalized to middle-aged and elderly people in China, and the impacts of solid fuels may vary among different populations. Second, we assessed whether solid fuel use was based on self-reported main fuel use for cooking rather than the participant’s actual exposure dose, and pollutant exposure dose could vary by the level and efficiency of ventilation, climate, and fuel properties; therefore, we could not find an association of fine PM or other pollutant compositions of solid fuel smoke with cognitive decline^43^. Third, we could not assess the exposure time of each participant^44^. Fourth, cognitive function in the study was assessed by self-rating scales, which are less than clinical diagnoses. Finally, interviewers collected information by a questionnaire so that recall bias of this study was possible.

## Conclusions

This study suggests that solid fuel use for cooking is associated with lower cognitive scores and that these scores were lower among female and rural users of solid fuel in the Chinese middle-aged and elderly populations. In addition, solid fuel use was related to accelerated decline in cognitive function, especially among female and older users. To our knowledge, our study supplemented evidence from a longitudinal study about the effects of solid fuel use on cognitive decline. Further studies including a more objective assessment of individual exposure to IAP from solid fuel burning are needed to confirm our findings.

## Data Availability

The CHARLS dataset can be applied for use by the web link: http://charls.pku.edu.cn/.
